# Transcriptome Analysis to Study the Molecular Response in the Gill and Hepatopancreas Tissues of *Macrobrachium nipponense* to Salinity Acclimation

**DOI:** 10.3389/fphys.2022.926885

**Published:** 2022-05-25

**Authors:** Cheng Xue, Kang Xu, Yiting Jin, Chao Bian, Shengming Sun

**Affiliations:** ^1^ Key Laboratory of Exploration and Utilization of Aquatic Genetic Resources, Shanghai Ocean University, Ministry of Education, Shanghai, China; ^2^ International Research Center for Marine Biosciences at Shanghai Ocean University, Ministry of Science and Technology, Shanghai, China; ^3^ Shenzhen Key Lab of Marine Genomics, Guangdong Provincial Key Lab of Molecular Breeding in Marine Economic Animals, BGI Academy of Marine Sciences, BGI Marine, Shenzhen, China

**Keywords:** *Macrobrachium* nipponense, transcriptome, salinity, crustaceans, carbonic anhydrase

## Abstract

*Macrobrachium nipponense* is an economically important prawn species and common in Chinese inland capture fisheries. During aquaculture, *M. nipponense* can survive under freshwater and low salinity conditions. The molecular mechanism underlying the response to salinity acclimation remains unclear in this species; thus, in this study, we used the Illumina RNA sequencing platform for transcriptome analyses of the gill and hepatopancreas tissues of *M. nipponense* exposed to salinity stress [0.4‰ (S0, control group), 6‰ (S6, low salinity group), and 12‰ (S12, high salinity group)]. Differentially expressed genes were identified, and several important salinity adaptation-related terms and signaling pathways were found to be enriched, such as “ion transport,” “oxidative phosphorylation,” and “glycometabolism.” Quantitative real-time PCR demonstrated the participation of 12 key genes in osmotic pressure regulation in *M. nipponense* under acute salinity stress. Further, the role of carbonic anhydrase in response to salinity acclimation was investigated by subjecting the gill tissues of *M. nipponense* to *in situ* hybridization. Collectively, the results reported herein enhance our understanding of the mechanisms via which *M. nipponense* adapts to changes in salinity.

## Introduction

The increase in inland salinity owing to human activities (Williams, 2001) has led to major economic, social, and environmental consequences. Inland saline water and saline–alkali land can be potentially used for aquaculture, serving as a source of revenue. Salinity is a key environmental factor in aquatic ecosystems and is known to influence the growth performance, survival, immune system, and respiratory and energy metabolism of crustaceans (Freire et al., 2011; Gao et al., 2016; Li et al., 2017; Koyama et al., 2018; Chen et al., 2019). Some aquatic crustaceans can tolerate a wide range of salinity levels; few previous studies have investigated the effects of salinity on the growth and development of *Macrobrachium nipponense* (Huang Y. H. et al., 2019), *M. rosenbergii* (Chand et al., 2015), *Penaeus monodon* (Le et al., 2009), and *Nephrops norvegicus* (Torres et al., 2011). It appears that aquatic crustaceans respond to changes in salinity via osmotic pressure regulation to maintain the balance of water and salt.

The adaptation of aquatic crustaceans to alterations in environmental salinity is a complex process and predominantly involves three types of regulatory pathways. First, osmotic pressure regulation is a pivotal process that controls the permeability and concentration of ions, such as Na^+^, K^+^, Ca^2+^, and Cl^−^, in plasma. Ion transporter proteins [Na^+^/K^+^-ATPase (NKA) and carbonic anhydrase (CA)] in the membranes of the epithelium and antennal glands are the main sites for regulating ion permeation in crustaceans in response to salinity acclimation (McNamara et al., 2015; Huang Y. et al., 2019). The CA enzyme appears to be a central molecular component in the adaptations to low salinity found in euryhaline crustaceans (Henry, 2001; Pongsomboon et al., 2009; Mitchell and Henry, 2014). Second, the energy channeled by crustaceans to respond to salinity acclimation leads to a significant reduction in growth, considering that the energy budget for osmotic adjustment is much higher (Ye et al., 2009; Ma et al., 2021). Therefore, fluctuations in water salinity seem to inhibit energy metabolism, which may cause oxidative damage (Choi et al., 2008). Finally, neuropeptides and their G protein-coupled receptors in the central nervous system regulate the physiological response of crustaceans to salinity acclimation (Sun et al., 2020; Réalis-Doyelle et al., 2021; Tong et al., 2022).

Transcriptome sequencing has been widely applied to develop molecular resources for non-model organisms with biological and economic importance (Riesgo et al., 2012; Sun et al., 2015; Liu et al., 2021). Transcriptome sequencing has been used to analyze the molecular mechanisms underlying the response to changes in salinity in many economically important species, such as *Scylla paramamosain*, *Litopenaeus vannamei*, *Eriocheir sinensis*, and *M. rosenbergii* (Zhang et al., 2015; Liu et al., 2016; Wang et al., 2018; Yang et al., 2019; Sun et al., 2020). To the best of our knowledge, although genomes information of the oriental freshwater prawn *M. nipponense* has been reported (Jin et al., 2021), salinity acclimation-related transcriptome data for gill and hepatopancreas tissues of the *M. nipponense* remain to be reported. As a member of euryhaline crustaceans, *M. nipponense* is primarily distributed in freshwater and low salt environments in Asia, and it is one of the most economically important aquaculture species in China, with annual production exceeding 200,000 tons and output reaching two billion RMB (Fu et al., 2012). Previous studies on *M. nipponense* have mainly focused on germplasm heredity, nutrition regulation, immunity performance, and resistance to hypoxia stress (Fu et al., 2004; Li et al., 2018; Sun et al., 2018; Zhao et al., 2018). To date, the molecular mechanisms via which *M. nipponense* responds to changes in salinity remain poorly understood.

Herein we performed transcriptome analysis of the hepatopancreas and gill tissues of *M. nipponense* cultivated at three different salinities using the Illumina platform to generate a *de novo* assembly. Our objectives were as follows: 1) construct hepatopancreas and gill tissue libraries of *M. nipponense* cultivated at different salinities; 2) identify differentially expressed genes (DEGs) and pathways that may play a key role in salinity acclimation in *M. nipponense*; and 3) validate target transcripts involved in osmotic pressure regulation, energy metabolism, and antioxidant defense that might have critical functions under conditions of acute salinity stress. The results of this study could improve our understanding of the molecular mechanisms used by *M. nipponense* to adapt to salinity acclimation, which is bound to enhance the sustainability of aquaculture production of *M. nipponense*.

## Materials and Methods

### Experimental Animals and Acclimation

Juvenile prawns (*M. nipponense*) were obtained from a farm in Shanghai (Qingpu) and acclimated to laboratory conditions for 14 days in freshwater (temperature, 24 ± 1°C; pH, 7.7 ± 0.6; dissolved oxygen, 6.5 ± 0.5 mg/L). Thereafter, 360 healthy prawns (2.15 ± 0.20 g wet weight) were randomly divided into 12 tanks (30 prawns/tank), and the tanks were randomly assigned to three groups (3 tanks/group). Salinity was gradually adjusted on the same day to reach target salinity levels for each group: S0 = ∼0‰ (control group), S6 = 6‰ ± 0.2‰ (low salinity), and S12 = 12‰ ± 0.2‰ (high salinity). Salinity and water quality were maintained as previously described (Huang Y. H. et al., 2019), and prawns were given commercial feed (Zhejiang Tongwei Feed Group CO, Ltd.) twice a day for 1 week at a ratio of 6%–8% of their body weight. After 4 weeks of acclimation, hepatopancreas and gill tissues were obtained from each treatment group in triplicates, rapidly frozen in liquid nitrogen, and stored at −80°C until needed.

### RNA Extraction, Library Construction, and Sequencing

The gill and hepatopancreas tissues were used for total RNA extraction. Briefly, total RNA was extracted using TRIzol (Invitrogen, CA, United States), as per manufacturer instructions. RNA quality and quantity were determined with a NanoDrop 2000 (Thermo Fisher Scientific Inc, United States) spectrophotometer; all OD_260_/OD_280_ values were in the range of 1.9–2.0. RNA integrity was assessed by 1% agarose gel electrophoresis, and 18 cDNA libraries were prepared using 2.5 μg total RNA, following the protocol of the Illumina TruSeq™ RNA Sample Preparation Kit. The libraries thus obtained were sequenced on Illumina HiSeq 2500 (2 × 150 bp read length; Illumina, Inc, San Diego, CA, United States) and paired-end reads were finally generated (Sun et al., 2020). The raw sequencing data have been deposited in the NCBI Sequence Read Archive (Accession no. SRP251206).

### *De Novo* Transcriptome Assembly and Gene Annotation

Raw data were processed to obtain clean reads, as follows: 1) joint sequences in reads were removed, 2) low-quality reads (bases with Q-value < 20) at the end of the sequence (3′-end) were eliminated, 3) reads with an N ratio of >10% were removed, and 4) reads containing adaptor sequences and sequences <75 bp after quality trimming were discarded.

The clean reads thus obtained were used for sequence assembly with Trinity v2.3.2 (Plymouth, MA, United States); default parameters were used for assembly generation, and the minimum contig length was 200 bp (Grabherr et al., 2011). All unigenes were annotated based on the following databases with a cut-off E-value of 10^–5^: NCBI non-redundant (Nr) (http://www.ncbi.nlm.nih.gov), Swiss-Prot (http://www.expasy.ch/sprot), Clusters of Orthologous Groups (COG) (http://www.ncbi.nlm.nih.gov/), and Kyoto Encyclopedia of Genes and Genomes (KEGG) (http://www.genome.jp/kegg). For Pfam domain/family annotation, predicted protein sequences were submitted to search against HMM profiles in the Pfam database v27.0 using HMMER v3.0 (Finn et al., 2017). Further, Blast2GO was used for gene ontology (GO) analysis (http://www.geneontology.org/), and COG classification and signal pathway annotation of unigenes were performed by conducting BLASTx searches against the COG and KEGG databases, respectively. Assembly metrics and annotation completeness were obtained using BUSCO 3.0.1 (Simão et al., 2015) with the arthropoda_odb9 dataset.

### Identification of DEGs and Enrichment Analysis

The expression of all unigenes was estimated by calculating read density as fragments per kilobase of transcript per million reads using the RSEM program (Li and Dewey, 2011). To identify DEGs, false discovery rate (FDR) ≤ 0.001 and two-fold change (log_2_ ratio) ≥ 1 or ≤ −1 defined significant differences in gene expression levels (Anders and Huber, 2010). Pearson correlation coefficient was determined as per the gene expression level of each sample, and hierarchical clustering was applied to classify samples with high similarity levels till all DEGs were clustered. Functional enrichment analysis of DEGs involved GO and KEGG pathway enrichment analyses, which were performed using Goatools (https://github.com/tanghaibao/GOatools) and KOBAS (http://kobas.cbi.pku.edu.cn/home.do), respectively (*p* ≤ 0.05).

### Identification of Simple Sequence Repeat Markers

The MIcroSAtellite search module (http://pgrc.ipk-gatersleben.de/misa/) was used to identify SSR markers and for primer design (Li et al., 2009). Mono-, di-, tri-, tetra-, penta-, and hexanucleotide repeat motifs were designed using default parameters; the minimum repeat numbers for these SSRs were 10, 6, 5, 5, 5, and 5, respectively.

### Quantitative Real-Time PCR (qPCR)

In order to observe expression trends of the DEGs, 12 DEGs with high expression levels from transcriptome were selected to observe genes expression profile according to acute salinity stress (18‰) using qPCR. RNA samples were extracted from hepatopancreas and gill tissue samples, which were collected at 3, 6, 12, 24, 48, and 96 h in *M. nipponense* responded to acute salinity stress. Specific primers were designed using Primer Premier 5.0. The *β*-actin gene served as an internal control for normalizing experimental results. Table 1 lists all gene primers.

**TABLE 1:** The specific primers used to in this study.

Primer Name	Sequence (5′→3′)
Carbonic anhydrase	F:TGGGTGTTTGACGGAGTGTTAAAGG
	R:CCTCTGCGGTGACGATGTTGAC
Heat shock protein 70	F: GCC​TCT​GCT​CAA​GCT​AGT​GT
	R: TGG​TGG​AAC​CTC​CAA​CAA​GG
Catalase	F:TCGTGGCTTCGCTGTCAAGTTC
	R:GGTGTGTTGCTGGATTCCTCTTCTG
copper/zinc superoxide dismutase	F:GGCTCATTACAACCCAGACGGATTC
	R:AGTTCCATCCTCACCGCTCTCG
manganese superoxide dismutase	F:TGTGGGTGTGAAAGGTTCTGGTTG
	R:GGGGTCCTGGTTTTGGCAAGTG
Glucose transporter 1	F: CCA​ACG​GGT​GTC​TGA​CAC​CTC​C
	R: GCA​CCT​ACT​GAA​AAT​AGA​GAC​A
glutathione peroxidase 3	F:AGAGGTTAATGGCGAGAAGGAACAC
	R:AGGGCGTTTGGATCAGCGAAAG
Hexokinase	F:CCACCCTCACTTCCACAATCTCATG
	R:GCAACAGCAGCAACCAAAGCAG
lactate dehydrogenase	F:CCAGAGGAGTGTGTATGCGGTTTC
	R:GTTGGTTCTTCTCGGCGTCTGTC
6-phosphofructokinase	F:GCTCACTTGCCTGTGGATCAGTTAG
	R:ATCTTCGCCGTCCTCTTCCTCTG
pyruvate dehydrogenase	F:ACAACAAGAGTAGCAGCAGGTCAAC
	R:TTCATCCCGCTCCATTTCTTCATCC
Na+/K + ATPase	F:CAGCCCAAGACGACATTCCCATC
	R:GTCACCGCAAGCCAATTCAACAC
β-actin	F:TATGCACTTCCTCATGCCAT
	R:AGGAGGCGGCAGTGGTCAT

First-stranded cDNA was synthesized from 1 μg RNA using the PrimeScript^®^ RT reagent kit (Takara, DRR037A, Dalian, China). qPCR was performed using Platinum SYBR Green qPCR SuperMix-UDG (Invitrogen, C11744-500, CA, United States); the 20-μL reaction mixture consisted of 10 μL buffer (qPCR SYBR Green Master Mix), 0.4 μL of forward and reverse primers each, and 9.2 μL template cDNA. The amplification cycle comprised the following steps: 40 cycles of 95°C for 30 s, 95°C for 10 s, and 60°C for 30 s *β*-actin served as the internal reference. The efficiency of amplification for each primer was estimated by constructing a standard curve using serial dilutions of pure cDNA samples; the efficiency values ranged from 0.9 to 1.02. The 2^−∆∆CT^ method was used to calculate relative expression levels (Livak and Schmittgen, 2001).

### *In Situ* Hybridization

*In situ* hybridization was performed to analyze the CA mRNA in the gill, which showed the highest expression level in those two tissues by qPCR analysis. *M. nipponense* gill tissue samples were obtained as described earlier, fixed in 4% paraformaldehyde in PBS, and incubated at 4°C overnight. The samples were then analyzed using *in situ* hybridization, according to our previous study (Sun et al., 2016). The anti-sense and sense probes of CISH (Chromogenic *in-situ* hybridization) study with DIG signal were designed by Primer 5 software, and synthesized by Shanghai Sangon Biotech Company. *In situ* hybridization experiments were performed in triplicate for each tissue. Slides were examined under light microscope for evaluation.

### Statistical Analysis

All data are presented as means ± standard error (SM). One-way analysis of variance (ANOVA) and Student’s t-tests were used to determine whether data were statistically significant (*p* < 0.05) between the control and the treatment groups. And the Dunn-Bonferroni post hoc method following a significant Kruskal–Wallis test was used when the data distribution was skewed.

## Results

### Transcriptome Sequencing and *De Novo* Assembly

On constructing and sequencing the 18 libraries from the three groups, we obtained approximately 790,425,774 raw reads and 118,563,866,100 raw bases, respectively. Analysis using the BUSCO pipeline indicated that >92% arthropoda orthologs were present in the assembled transcriptome [93.2% complete BUSCOs (C)]. After removing adaptor sequences, ambiguous ‘N’ nucleotides, and low-quality sequences, 730,374,278 clean reads, representing 108,911,319,233 clean nucleotides, were obtained. The average Q30 percentage and GC content were 94.66% and 44.59%, respectively, indicating the high accuracy of our transcriptome data (Table 2). In total, 162,250 unigenes were obtained from the combined transcripts, with the total length being 187,598,373 bp. Among these unigenes, 52,154 (32.14%) were 1–600 bp, 61,901 (38.14%) were 601–1200 bp, and 48,195 (29.72%) were >1200 bp. Supplementary Tables S1, S2 provide an overview of assembly results. Supplementary Figure S1 shows specific fragment distribution.

**TABLE 2:** Basic statistics of RNA-seq reads in *M. nipponense*.

Sample	Raw Reads	Raw Bases	Clean Reads	Clean Bases	Q20%^a^	Q30%^b^	GC%^c^
LG-1	39,171,432	5,875,714,800	35,393,414	5,275,374,022	98.52	94.95	44.82
LG-2	46,201,618	6,930,242,700	40,744,942	6,065,221,795	98.46	94.76	44.77
LG-3	47,266,458	7,089,968,700	42,426,326	6,321,349,979	98.5	94.9	44.6
HG-1	40,253,752	6,038,062,800	37,071,372	5,531,352,170	98.57	95.01	42.04
HG-2	59,588,288	8,938,243,200	54,053,198	8,060,452,409	98.62	95.17	42.89
HG-3	41,314,824	6,197,223,600	38,706,680	5,778,650,912	98.68	95.38	44.7
FG-1	40,668,266	6,100,239,900	37,999,698	5,657,273,336	98	93.61	44.27
FG-2	53,989,276	8,098,391,400	50,753,610	7,557,280,709	98.06	93.76	44.43
FG-3	41,389,072	6,208,360,800	38,618,716	5,749,922,485	98.11	93.9	43.8
LH-1	41,580,366	6,237,054,900	38,764,410	5,786,335,185	98.35	94.48	46.32
LH-2	38,308,492	5,746,273,800	35,572,040	5,308,698,607	98.57	95.07	46.46
LH-3	40,942,340	6,141,351,000	37,801,184	5,639,162,660	98.54	94.99	46.1
HH-1	38,824,410	5,823,661,500	36,002,966	5,377,877,295	98.62	95.17	43.98
HH-2	45,160,524	6,774,078,600	42,106,088	6,290,257,258	98.7	95.41	44.46
HH-3	44,719,422	6,707,913,300	40,445,100	6,038,081,919	98.58	95.08	43.74
FH-1	37,313,474	5,597,021,100	35,177,300	5,241,056,393	98.13	93.96	44.2
FH-2	49,288,664	7,393,299,600	46,567,284	6,944,014,083	98.12	93.92	45.86
FH-3	44,445,096	6,666,764,400	42,169,950	6,288,958,016	98.28	94.36	45.09
Sum	790,425,774	118,563,866,100	730,374,278	108,911,319,233	/	/	/
Average	43,912,543	6,586,881,450	40,576,348	6,050,628,846	98.42	94.66	44.59

LG, low-salinity gill tissue; HG, high-salinity gill tissue; FG, freshwater gill tissue; LH: low-salinityhepatopancreas tissue; HH: high-salinity hepatopancrea tissue; FH, freshwater hepatopancrea tissue.

a Q20%, percentage of bases with Phred value > 20.

b Q30%, percent of bases with Phred value > 30.

c GC%, percentage of G and C bases among total bases.

### Gene Annotation and COG Assignment

**FIGURE 1:**
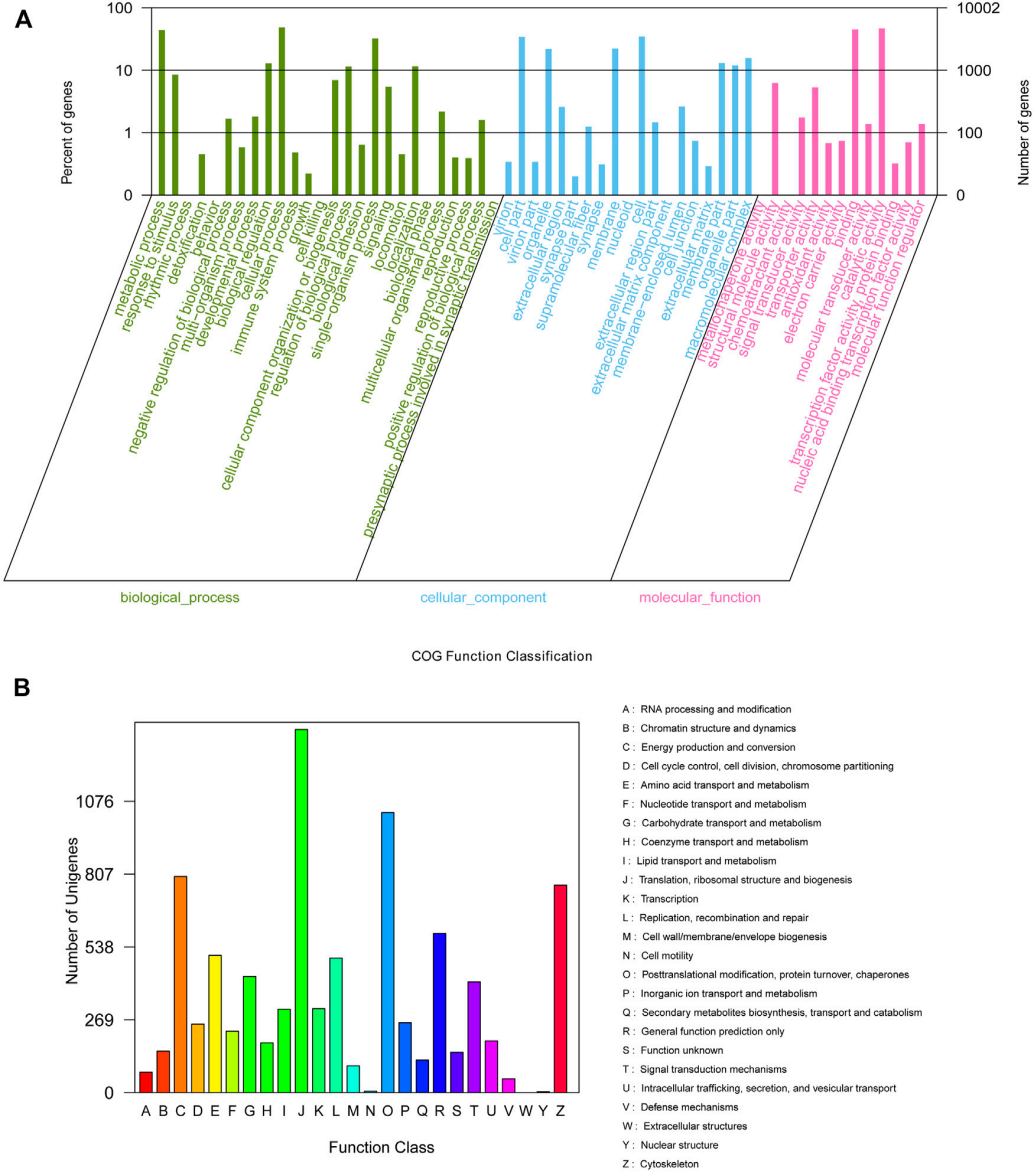
GO **(A)** categorization and COG **(B)** functional classification of assembled unigenes.

For the unigene sequences obtained by splicing, BLASTx comparison (BLAST+ 2.7.1, E-value < 10^–5^) was used for annotation against the COG, GO, KEGG, Swiss-Prot, and NCBI NR databases. In total, 162,250 unigenes were searched; of them, 51,172 (31.54%), 34,276 (21.13%), 10,002 (6.16%), 27,554 (16.98%), and 16,078 (9.89%) were annotated using the Nr, Swiss-Prot, GO, KEGG, and COG databases, respectively (Supplementary Table S3). On GO analysis, 10,002 unigenes were enriched into 58 functional subgroups. Further, based on COG analysis, 8,755 unigenes were allocated to 25 COGs (Figure 1A, B).

### DEGs

**FIGURE 2:**
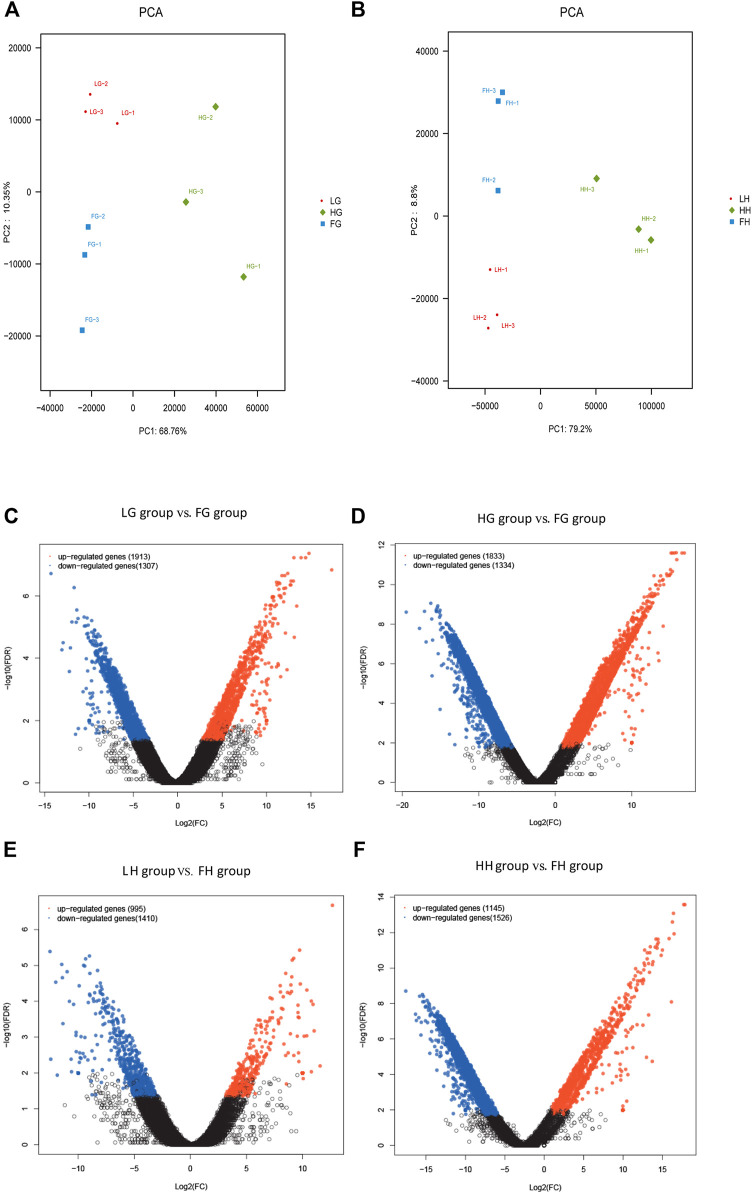
Analysis of DEGs. **(A,B)** Principal component analysis plot of transcriptome data obtained on assessing the gill and hepatopancreas tissues of *M. nipponense*. Analysis of DEGs identified in the gill and hepatopancreas tissues from the control *vs* low salinity group **(C,E)** and the control *vs* high salinity group **(D & F)**. Up- and downregulated DEGs are shown in red and blue, respectively. The X- and *Y*-axes show the log_2_-fold change and log_10_ *p*-value of normalized expression level (fragments per kilobase of transcript per million mapped reads) of a gene between the aforementioned groups, respectively.

The expression levels of DEGs in the six groups were evaluated using FPKM values: low-salinity gill tissue (LG), high-salinity gill tissue (HG), freshwater gill tissue (FG), low-salinity hepatopancreas tissue (LH), high-salinity hepatopancreas tissue (HH), and freshwater hepatopancreas tissue (FH). All DEGs with the absolute value of log_2_ ratio ≥1 and FDR ≤0.001 are shown in Figure 2. Overall, 3,220 and 3,167 DEGs were detected on comparing LG *vs* FG (1,913 up- and 1,307 down-regulated genes) and HG *vs* FG (1,833 up- and 1,334 down-regulated genes), respectively, and 2,405 and 2,671 DEGs were detected on comparing LH *vs* FH (955 up- and 1,410 down-regulated genes) and HH *vs* FH (1,145 up- and 1,526 down-regulated genes), respectively.

### Functional Annotation of DEGs

**FIGURE 3:**
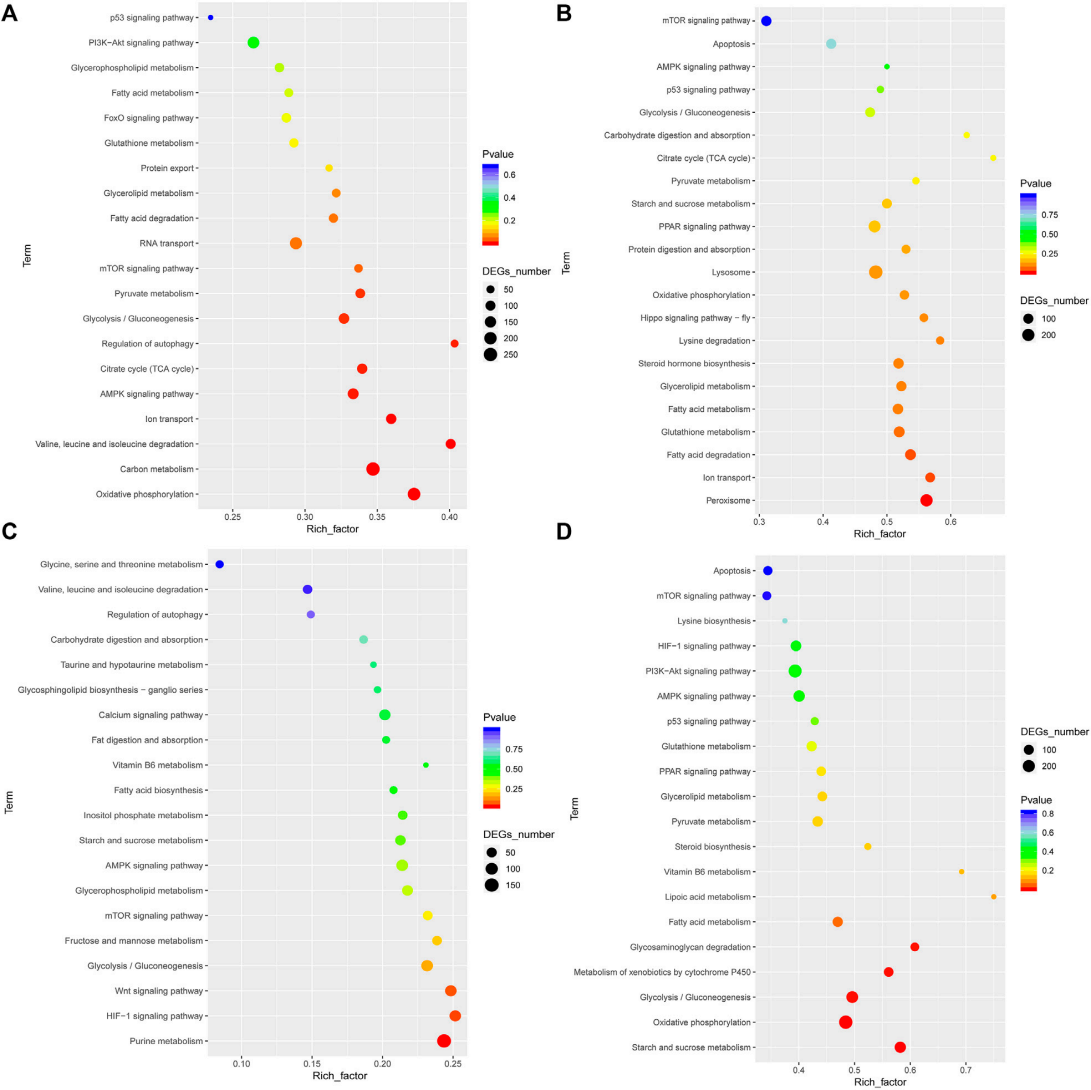
Significant KEGG pathway classifications of DEGs in the gill and hepatopancreas tissues of *M. nipponense* are shown for the control *vs* low salinity group **(A,C)** and for the control *vs* high salinity group **(B,D)**.

To analyze the functions of unigenes, GO assignments were made (*p* < 0.05 and FDR <0.001). DEGs were subjected to GO enrichment analyses. Of the 3,815 GO terms, 2,114 (55.41%) terms were involved in biological processes, 543 (14.23%) in cellular components, and 1,158 (30.35%) in molecular functions (Supplementary Table S4). On comparing LH *vs* FH and HH *vs* FH, the most significantly enriched GO terms were “cellular process,” “metabolic process,” “cell,” “binding,” “transport activity,” and “catalytic activity” (Supplementary Figure S2). Further, on comparing LG *vs* FG and HG *vs* FG, the most significantly enriched GO terms were “cellular process,” “metabolic process,” “cell part,” “catalytic activity, “cell,” and “binding” (Supplementary Figure S2). To analyze the involvement of DEGs in various signaling pathways, unigenes were annotated using the KEGG database. KEGG pathways are listed in Supplementary Figure S3. In LG *vs* FG, the significantly enriched pathways included “pyruvate,” “glycolysis/gluconeogenesis,” “TCA cycle,” “ion transport,” and “AMPK signaling pathway.” In HG *vs* FG, the significantly enriched pathways included “ion transport,” “p53 signaling pathway,” “oxidative phosphorylation,” and “apoptosis.” Similarly, in LH *vs* FH and HH *vs* FH, the significantly enriched pathways were associated with energy metabolism (Figure 3).

### Analysis of SSRs

In total, 73,804 SSRs were identified. All SSRs were identified in 194,085 unigenes, and 14,923 unigenes contained >1 SSR (Supplementary Table S5). Of the 73,804 SSR motifs identified in *M. nipponense*, the largest fraction consisted of mononucleotides (30,063), followed by di- (28,021) and trinucleotides (14,273). A/T (29,334) was the most common type of mononucleotide repeat motif, followed by C/G (620), and AG/CT (18,107) was the most common type of dinucleotide repeat motif, followed by AT/AT (4,977) and AC/GT (4,819) (Supplementary Figure S3).

### qPCR Validation

**FIGURE 4:**
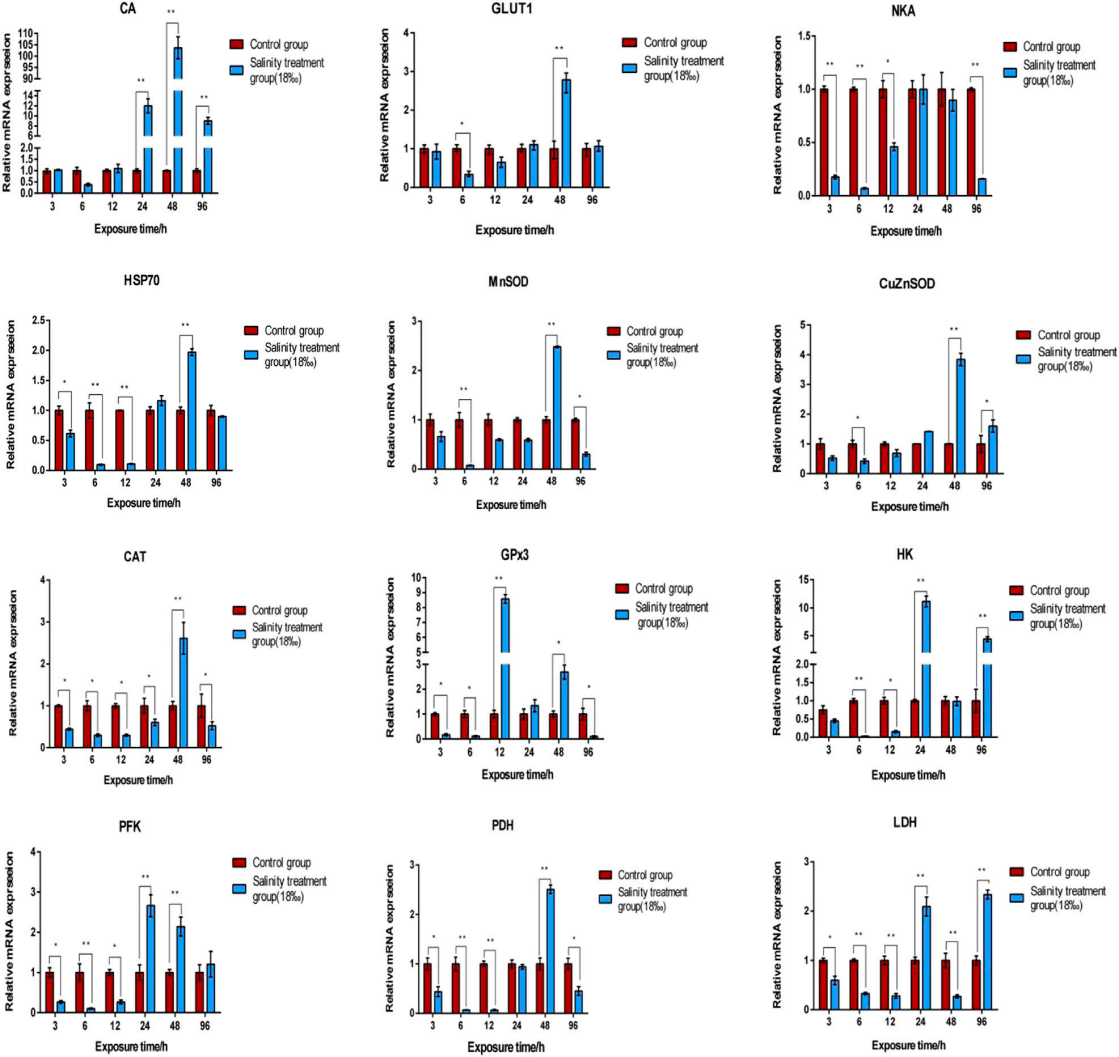
Validation of the expression profiles of 12 DEGs identified in the gill tissue of *M. nipponense* using qPCR. Log-fold changes are expressed as the ratio of gene expression after normalization to *β*-actin expression levels. Data are shown as mean ± SE (standard error) of tissues in three separate individuals. *indicates significant difference (*p* < 0.05), ^∗∗^indicates extremely significant difference (*p* < 0.01).

**FIGURE 5:**
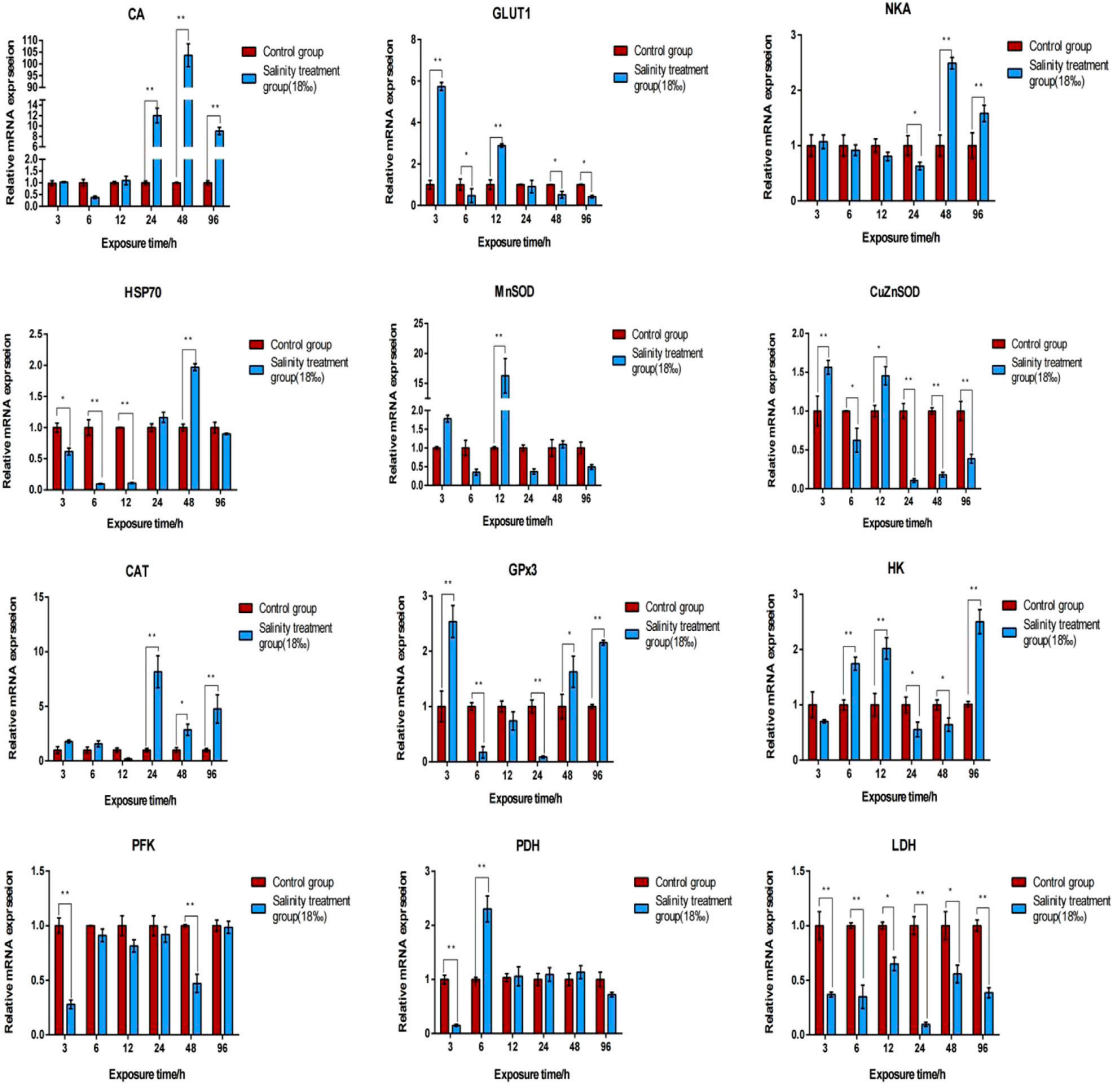
Validation of the expression profiles of 12 DEGs identified in the hepatopancreas tissue of *M. nipponense* using qPCR. Log-fold changes are expressed as the ratio of gene expression after normalization to *β*-actin expression levels. Data are shown as mean ± SE (standard error) of tissues in three separate individuals. *indicates significant difference (*p* < 0.05), ^∗∗^indicates extremely significant difference (*p* < 0.01).

Table S6lists DEGs identified across all comparative groups. To investigate the gene expression profile in the gill and hepatopancreas tissues of *M. nipponense* in response to 96-h acute salinity stress (18‰), twelve salinity adaptation-related genes were chosen: CA, NKA, catalase (CAT), Cu/Zn superoxide dismutase (Cu/ZnSOD), Mn superoxide dismutase (MnSOD), glucose transporter (GLUT1), glutathione peroxidase 3 (GPx3), hexokinase (HK), lactate dehydrogenase (LDH), phosphofructose kinase (PFK), pyruvate dehydrogenase (PDH), heat shock protein 70 (HSP70). qPCR results show the effects of salinity on the expression of these genes in the gill (Figure 4) and hepatopancreas (Figure 5) tissues of *M. nipponense*, respectively. We found that gens involved in antioxidant, glycolysis and osmotic reglution play important role in response acute salinity stress, especially in CA gene.

### Localization of CA mRNA

**FIGURE 6:**
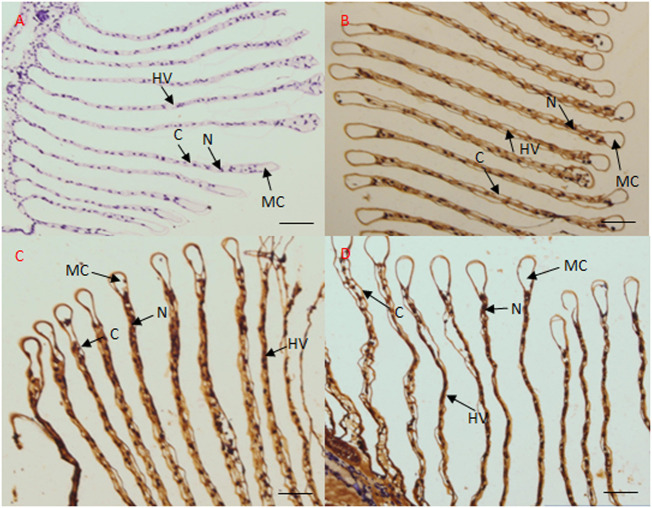
Localization of CA transcripts in the gill tissue of *M. nipponense* by *in situ* hybridization. Sense probes were used as negative controls, which generated no signal **(A)**. In the gill tissue, CA transcripts mainly localized to the epithelial cell nuclei and hemolymph vessel under freshwater **(B)**, low salinity **(C)**, and high salinity **(D)** conditions. MC, marginal channel; **(C)** cuticle; N, epithelial cell nuclei; HV, hemolymph vessel. Scale bar: 50 μm.

To further confirm this finding, *in situ* hybridization was performed using frozen gill and hepatopancreas tissues. In case of the gill tissue, no signal was observed when the negative control sense strand probe (Figure 6A) was hybridized with the sense CA probe; however, in response to salinity acclimation, the antisense probe generated a positive signal in the epithelial cell nuclei, cytoplasm, and hemolymph vessel (Figure 6B). Further, in response to high salinity, the antisense probe yielded a signal in the epithelial cell nuclei (Figures 6C,D).

## Discussion

Euryhaline crustaceans are a key model organism for investigating fundamental and evolutionarily conserved processes. The chromosome-level genome assembly of *M. nipponense* was recently reported; the total assembled genome size was approximately 4.5 Gb, and a scaffold N50 of 86.8 Mb was produced using the Illumina platform (Jin et al., 2021). Our previous study used the Illumina platform to conduct gene expression profile analyses so as to identify neuropeptides and G protein-coupled receptors from eyestalk tissues of prawns exposed to salinity stress (Sun et al., 2020). Further, a recent study evaluated the effects of salinity, photoperiod, and light spectrum on larval survival, growth, and related enzyme activities in the giant freshwater prawn *M. rosenbergii* (Wei et al., 2021). However, the molecular responses in the gill and hepatopancreas tissues of *M. nipponense* in response to salinity acclimation remain unknown. Few previous studies have attempted to investigate osmotic adaptive response of estuarine crustaceans by mimicking salinity stress in laboratory environments (Li et al., 2015; Hudson et al., 2018; Liu et al., 2022). In case of the oriental river prawn, 14‰ is the tolerable salinity level for normal physiological activities (Huang Y. H. et al., 2019), with the gills and hepatopancreas being the core metabolic tissues and the main sites of ion transport (Pierrot et al., 1995; Gao et al., 2012). Thus, in this study, we identified DEGs in the gill and hepatopancreas tissues of *M. nipponense* subjected to acute salinity stress.

### Enrichment Analysis to Explore Major Biological Associations

Crustaceans tackle changes in environmental salinity, for example, via osmotic pressure regulation, acid–base balance regulation, and respiratory metabolism (Chen and Lin, 1995; Long et al., 2017; Chen et al., 2019). To understand the molecular regulation mechanism of *M. nipponense* in response to salinity acclimation, transcriptome data were obtained by assessing the gill and hepatopancreas tissues of prawns cultured under S0 (freshwater), S6 (low salinity), and S12 (high salinity) conditions. GO and KEGG pathway enrichment analyses led to the identification of differentially enriched pathways, such as “glycolysis/gluconeogenesis,” “TCA cycle,” and “fatty acid metabolism.” Crustaceans, such as Chinese shrimp *Fenneropenaeus chinensis* (Li et al., 2019) and Pacific white shrimp *L. vannamei* (Chen et al., 2015), evidently increase their dependence on aerobic metabolism for fueling osmoregulation. It appears that glycolipid metabolism reduces osmotic stress in crustaceans by supplying extra energy or adjusting the membrane structure to facilitate osmoregulation in organs such as the gills. Other important GO terms identified in this study included “oxidative phosphorylation” and “peroxisome.” These findings indicated that high salinity induces oxidative stress, maybe leading to oxidative damage and apoptosis in crustaceans (Rivera-Ingraham et al., 2016; Huang et al., 2020).

### Osmoregulation

Decapod crustaceans show variable degrees of euryhalinity and osmoregulatory capacity, and they respond to changes in salinity through anisosmotic extracellular regulation and/or cell volume regulation (Cuenca et al., 2021). Osmotic pressure regulation is an important method for crustaceans to adapt to changes in salinity (Malik and Kim, 2021). NKA and CA are widely known ion transporters that play a key role in osmotic pressure balance in crustaceans. Different ions are transported through different ion transport channels (Huang Y. et al., 2019). Herein our transcriptome analysis revealed that in comparison to the control groups, the mRNA expression levels of NKA and CA were significantly up-regulated in the gill and hepatopancreas tissues of *M. nipponense* in the salinity acclimation groups (HG *vs* FG, LG *vs* FG, HH *vs* FH, and LH *vs* FH). qPCR results indicated that the expression levels of NKA and CA in the gill and hepatopancreas tissues of *M. nipponense* were significantly upregulated under acute salinity stress conditions as compared to those under freshwater conditions. Similar expression profiles have been reported for other crustaceans, such as *E. sinensis* and *L. vannamei* (Mao et al., 2014; Liu et al., 2015). Collectively, these data suggest that NKA and CA play a key role in osmotic pressure regulation in *M. nipponense* under conditions of acute salinity stress and chronic salinity acclimation. In addition, we found that CA was mainly localized in the epithelial cell nuclei and hemolymph vessels in the gill tissue, suggesting that Carbonic anhydrase (CA) is a ubiquitous enzyme involved in acid-base regulation and osmoregulation in gill tissue of crustacean, which was similar with previous studies in fish (Gilmour, 2012; Ferreira-Martins et al., 2016; Kumar et al., 2020). Thus, we believe that the CA gene can serve as a molecular indicator of acute salinity stress in *M. nipponense* at specific time points.

### Energy Metabolism

Ion transport and ion channel proteins participate in osmotic pressure regulation, which requires abundant energy (Tseng and Hwang, 2008). Glucose metabolism, which involves glycolysis, followed by the TCA cycle and electron transport chain, plays a chief role in supplying energy for osmotic pressure regulation (De Boeck et al., 2000). Little is known of the properties and variation of anaerobic and aerobic metabolism in tissues during osmoregulation. In this study, transcriptome sequencing results indicated that the expression of HK, PFK, PK, PDH, LDH, and GLUT1 was upregulated in the gill and hepatopancreas tissues of *M. nipponense* under low salinity conditions. Under high salinity conditions, the expression of citrate synthase and cytochrome C oxidase was downregulated and that of other glycolysis-related enzymes was upregulated. Moreover, our results demonstrated that the expression of the rate-limiting enzymes PFK and LDH was markedly upregulated under high salinity conditions (Li et al., 2019). It appears that under conditions of acute high salinity stress, glycolysis is the most primitive method for organisms such as prawns and oysters to acquire energy (Chen et al., 2022). However, osmoregulation at high salinity levels might disrupt glucose metabolism. Therefore, exposing prawn to very high salinity conditions, such as during stock enhancement, should be avoided.

### Antioxidant System

Changes in the water environment can induce oxidative stress and antioxidant response in aquatic organisms (Wang et al., 2009; Wang et al., 2020). In response to salinity stress, shrimps produce reactive oxygen species (ROS), which can destroy cells, cause morphological changes in the hepatopancreas, and induce the expression of antioxidant enzymes and proteins (Paital and Chainy, 2010). ROS are thus continuously removed by the antioxidant enzyme defense system, which mainly comprises SOD, CAT, and GPx (Cheng and Chen, 2000; Pan et al., 2005). The process of removing ROS is as follows: SOD catalyzes O_2_^−^ disproportionation into H_2_O_2_ and H_2_O, and CAT then decomposes H_2_O_2_ into H_2_O and O_2_, resulting in effective detoxification (Zhang et al., 2007). In the antioxidant stress response, HSPs catalyze the conversion of ROS by activating endogenous peroxidase (Shi et al., 2016). In this study, transcriptome analyses indicated that in comparison to the control groups, the expression of SODs (Cu/ZnSOD and MnSOD) was downregulated in the salinity acclimation groups, while that of CAT, GPx3, and HSP70 was upregulated. Further, we found that 96-h acute salinity stress inhibited the mRNA expression of MnSOD, CAT, and GPx3 in the gill tissue of *M. nipponense*; however, in the hepatopancreas tissue, an increase followed by a decrease was observed in the mRNA expression of CAT and GPx3. This could have been a mechanism to activate antioxidant and heat stress responses and to reduce oxidative damage. Altogether, these findings suggested that salinity stress modulates oxidative stress and antioxidant defenses in *M. nipponense* in a tissue-specific manner, which is similar to the results reported for *S. serrata* (Paital and Chainy, 2010).

To summarize, we performed transcriptome analyses to investigate the molecular response of the gill and hepatopancreas tissues of *M. nipponense* to salinity acclimation. Several DEGs and core pathways involved in, for example, osmotic pressure regulation and energy metabolism, were identified. Further, based on CA localization results obtained on performing *in situ* hybridization, we believe that the CA gene can be used as a molecular indicator of acute salinity stress in *M. nipponense*. Future studies are warranted to further understand pertinent mechanisms so as to develop new methods to enhance the survival rate of *M. nipponense* and improve brackish water aquaculture.

## Data Availability

The datasets presented in this study can be found in online repositories. The names of the repository/repositories and accession number(s) can be found in the article/Supplementary Material.

## References

[B1] Anders S. Huber W. (2010). Differential Expression Analysis for Sequence Count Data. Genome. Biol. 11, R106. 10.1186/gb-2010-11-10-r106 20979621 PMC3218662

[B2] Chand B. K. Trivedi R. K. Dubey S. K. Rout S. K. Beg M. M. Das U. K. (2015). Effect of salinity on survival and growth of giant freshwater prawn Macrobrachium rosenbergii (de Man). Aquac. Rep. 2, 26–33. 10.1016/j.aqrep.2015.05.002

[B3] Chen J. C. Lin C. Y. (1995). Responses of Oxygen Consumption, Ammonia-N Excretion and Urea-N Excretion of *Penaeus Chinensis* Exposed to Ambient Ammonia at Different Salinity and pH Levels. Aquaculture 136 (3-4), 243–255. 10.1016/0044-8486(95)01060-2

[B4] Chen K. Li E. Li T. Xu C. Wang X. Lin H. (2015). Transcriptome and Molecular Pathway Analysis of the Hepatopancreas in the Pacific White Shrimp *Litopenaeus Vannamei* under Chronic Low-Salinity Stress. PLoS One 10 (7), e0131503. 10.1371/journal.pone.0131503 26147449 PMC4492601

[B5] Chen L. Yu F. Shi H. Wang Q. Xue Y. Xue C. (2022). Effect of Salinity Stress on Respiratory Metabolism, Glycolysis, Lipolysis, and Apoptosis in Pacific Oyster ( Crassostrea gigas ) during Depuration Stage. J. Sci. Food. Agric. 102 (5), 2003–2011. 10.1002/jsfa.11539 34537961

[B6] Chen X. Chen J. Shen Y. Bi Y. Hou W. Pan G. (2019). Transcriptional Responses to Low-Salinity Stress in the Gills of Adult Female *Portunus Trituberculatus* . Comp. Biochem. Physiology Part D Genomics Proteomics 29, 86–94. 10.1016/j.cbd.2018.11.001 30463042

[B7] Cheng W. Chen J.-C. (2000). Effects of pH, Temperature and Salinity on Immune Parameters of the Freshwater Prawn Macrobrachium Rosenbergii. Fish Shellfish Immunol. 10, 387–391. 10.1006/fsim.2000.0264 10938749

[B8] Choi C. Y. An K. W. An M. I. (2008). Molecular Characterization and mRNA Expression of Glutathione Peroxidase and Glutathione S-Transferase during Osmotic Stress in Olive Flounder (*Paralichthys olivaceus*). Comp. Biochem. Physiology Part A Mol. Integr. Physiology 149 (3), 330–337. 10.1016/j.cbpa.2008.01.013 18302988

[B9] Cuenca A. L. R. Souza M. M. Freire C. A. (2021). Osmoregulatory Power Influences Tissue Ionic Composition after Salinity Acclimation in Aquatic Decapods. Comp. Biochem. Physiology Part A Mol. Integr. Physiology 259, 111001. 10.1016/j.cbpa.2021.111001 34098129

[B10] De Boeck G. Vlaeminck A. Van der Linden A. Blust R. (2000). The Energy Metabolism of Common Carp (*Cyprinus carpio*) when Exposed to Salt Stress: an Increase in Energy Expenditure or Effects of Starvation? Physiological Biochem. Zoology 73 (1), 102–111. 10.1086/316717 10685912

[B11] Ferreira-Martins D. McCormick S. D. Campos A. Lopes-Marques M. Osório H. Coimbra J. (2016). A Cytosolic Carbonic Anhydrase Molecular Switch Occurs in the Gills of Metamorphic Sea Lamprey. Sci. Rep. 6, 33954. 10.1038/srep33954 27703170 PMC5050428

[B12] Finn R. D. Attwood T. K. Babbitt P. C. Bateman A. Bork P. Bridge A. J. (2017). InterPro in 2017-beyond Protein Family and Domain Annotations. Nucleic. acids. Res. 45 (D1), D190–D199. 10.1093/nar/gkw1107 27899635 PMC5210578

[B13] Freire C. A. Togni V. G. Hermes-Lima M. (2011). Responses of Free Radical Metabolism to Air Exposure or Salinity Stress, in Crabs (*Callinectes Danae* and *C. ornatus*) with Different Estuarine Distributions. Comp. Biochem. Physiology Part A Mol. Integr. Physiology 160 (2), 291–300. 10.1016/j.cbpa.2011.06.024 21742051

[B14] Fu H. Gong Y. Wu Y. Xu P. Wu C. (2004). Artificial Interspecific Hybridization between *Macrobrachium* Species. Aquaculture 232 (1-4), 215–223. 10.1016/j.aquaculture.2003.08.002

[B15] Fu H. Jiang S. Xiong Y. (2012). Current Status and Prospects of Farming the Giant River Prawn (*Macrobrachium Rosenbergii*) and the Oriental River Prawn (*Macrobrachium Nipponense*) in China. Aquac. Res. 43 (7), 993–998. 10.1111/j.1365-2109.2011.03085.x

[B16] Gao W. Tan B. Mai K. Chi S. Liu H. Dong X. (2012). Profiling of Differentially Expressed Genes in Hepatopancreas of White Shrimp (*Litopenaeus Vannamei*) Exposed to Long-Term Low Salinity Stress. Aquaculture 364-365, 186–191. 10.1016/j.aquaculture.2012.08.024

[B17] Gao W. Tian L. Huang T. Yao M. Hu W. Xu Q. (2016). Effect of Salinity on the Growth Performance, Osmolarity and Metabolism-Related Gene Expression in White Shrimp Litopenaeus Vannamei. Aquac. Rep. 4, 125–129. 10.1016/j.aqrep.2016.09.001

[B18] Gilmour K. M. (2012). New Insights into the Many Functions of Carbonic Anhydrase in Fish Gills. Respir. Physiology Neurobiol. 184 (3), 223–230. 10.1016/j.resp.2012.06.001 22706265

[B19] Grabherr M. G. Haas B. J. Yassour M. Levin J. Z. Thompson D. A. Amit I. (2011). Full-length Transcriptome Assembly from RNA-Seq Data without a Reference Genome. Nat. Biotechnol. 29 (7), 644–652. 10.1038/nbt.1883 21572440 PMC3571712

[B20] Henry R. P. (2001). Environmentally Mediated Carbonic Anhydrase Induction in the Gills of Euryhaline Crustaceans. J. Exp. Biol. 204 (5), 991–1002. 10.1242/jeb.204.5.991 11171422

[B21] Huang Y. H. Zhang M. Li Y. M. Wu D. L. Liu Z. Q. Jiang Q. C. (2019a). Effects of Salinity Acclimation on the Growth Performance, Osmoregulation and Energy Metabolism of the Oriental River Prawn, *Macrobrachium Nipponense* (De Haan). Aquac. Res. 50, 685–693. 10.1111/are.13950

[B22] Huang Y. Liu Z. Li Y. Wu D. Zhang M. Zhao Y. (2019b). Cloning and Characterisation of Na+/K+-ATPase and Carbonic Anhydrase from Oriental River Prawn Macrobrachium Nipponense. Int. J. Biol. Macromol. 129, 809–817. 10.1016/j.ijbiomac.2019.02.098 30784852

[B23] Huang Y. Wu D. Li Y. Chen Q. Zhao Y. (2020). Characterization and Expression of Arginine Kinase 2 from *Macrobrachium Nipponense* in Response to Salinity Stress. Dev. Comp. Immunol. 113, 103804. 10.1016/j.dci.2020.103804 32738337

[B24] Hudson D. M. Sexton D. J. Wint D. Capizzano C. Crivello J. F. (2018). Physiological and Behavioral Response of the Asian Shore crab,Hemigrapsus Sanguineus, to Salinity: Implications for Estuarine Distribution and Invasion. Peer. J. 6, e5446. 10.7717/peerj.5446 30128204 PMC6097503

[B25] Jin S. Bian C. Jiang S. Han K. Xiong Y. Zhang W. (2021). A Chromosome-Level Genome Assembly of the Oriental River Prawn, *Macrobrachium Nipponense* . Gigascience 10 (1), giaa160. 10.1093/gigascience/giaa160 33459341 PMC7812440

[B26] Koyama H. Mizusawa N. Hoashi M. Tan E. Yasumoto K. Jimbo M. (2018). Changes in Free Amino Acid Concentrations and Associated Gene Expression Profiles in the Abdominal Muscle of Kuruma Shrimp (*Marsupenaeus japonicus*) Acclimated at Different Salinities. J. Exp. Biol. 221, jeb168997. 10.1242/jeb.168997 29674374

[B27] Kumar M. Varghese T. Sahu N. P. Gupta G. Dasgupta S. (2020). Pseudobranch Mimics Gill in Expressing Na^+^K^+^-ATPase 1 α-subunit and Carbonic Anhydrase in Concert with H^+^-ATPase in Adult Hilsa (Tenualosa Ilisha) during River Migration. Fish. Physiol. Biochem. 46, 725–738. 10.1007/s10695-019-00746-y 31848826

[B28] Li B. Dewey C. N. (2011). RSEM: Accurate Transcript Quantification from RNA-Seq Data with or without a Reference Genome. Bmc. Bioinforma. 12, 323. 10.1186/1471-2105-12-323 PMC3163565 21816040

[B29] Li E. Wang X. Chen K. Xu C. Qin J. G. Chen L. (2017). Physiological Change and Nutritional Requirement of Pacific White shrimpLitopenaeus Vannameiat Low Salinity. Rev. Aquacult. 9 (1), 57–75. 10.1111/raq.12104

[B30] Li J. Ma P. Liu P. Chen P. Li J. (2015). The Roles of Na^+^/K^+^-ATPase α-subunit Gene from the Ridgetail White Prawn Exopalaemon Carinicauda in Response to Salinity Stresses. Fish Shellfish Immunol. 42 (2), 264–271. 10.1016/j.fsi.2014.10.043 25449370

[B31] Li J. Xu X. Li W. Zhang X. (2019). Linking Energy Metabolism and Locomotor Variation to Osmoregulation in Chinese Shrimp *Fenneropenaeus Chinensis* . Comp. Biochem. Physiology Part B Biochem. Mol. Biol. 234, 58–67. 10.1016/j.cbpb.2019.05.006 31077786

[B32] Li R. Li Y. Fang X. Yang H. Wang J. Kristiansen K. (2009). SNP Detection for Massively Parallel Whole-Genome Resequencing. Genome Res. 19 (6), 1124–1132. 10.1101/gr.088013.108 19420381 PMC2694485

[B33] Li Y. Fan B. Huang Y. Wu D. Zhang M. Zhao Y. (2018). Effects of Dietary Vitamin E on Reproductive Performance and Antioxidant Capacity of *Macrobrachium Nipponense* Female Shrimp. Aquacult Nutr. 24 (6), 1698–1708. 10.1111/anu.12804

[B34] Liu B. Gao Q. Liu B. Song C. Sun C. Liu M. (2022). Application of Transcriptome Analysis to Understand the Adverse Effects of Hypotonic Stress on Different Development Stages in the Giant Freshwater Prawn *Macrobrachium Rosenbergii* Post-larvae. Antioxidants 11 (3), 440. 10.3390/antiox11030440 35326091 PMC8944765

[B35] Liu M. Liu S. Hu Y. Pan L. (2015). Cloning and Expression Analysis of Two Carbonic Anhydrase Genes in White Shrimp *Litopenaeus Vannamei*, Induced by pH and Salinity Stresses. Aquaculture 448, 391–400. 10.1016/j.aquaculture.2015.04.038

[B36] Liu S. Chen G. Xu H. Zou W. Yan W. Wang Q. (2017). Transcriptome Analysis of Mud Crab ( Scylla Paramamosain ) Gills in Response to Mud Crab Reovirus (MCRV). Fish Shellfish Immunol. 60 (2), 545–553. 10.1016/j.fsi.2016.07.033 27492124

[B37] Liu Z. Li Y. Pérez E. Jiang Q. Chen Q. Jiao Y. (2021). Polystyrene Nanoplastic Induces Oxidative Stress, Immune Defense, and Glycometabolism Change in *Daphnia pulex*: Application of Transcriptome Profiling in Risk Assessment of Nanoplastics. J. Hazard. Mater. 402, 123778. 10.1016/j.jhazmat.2020.123778 33254789

[B38] Livak K. J. Schmittgen T. D. (2001). Analysis of Relative Gene Expression Data Using Real-Time Quantitative PCR and the 2−ΔΔCT Method. Methods 25, 402–408. 10.1006/meth.2001.1262 11846609

[B39] Long X. Wu X. Zhao L. Ye H. Cheng Y. Zeng C. (2017). Effects of Salinity on Gonadal Development, Osmoregulation and Metabolism of Adult Male Chinese Mitten Crab, *Eriocheir Sinensis* . PLoS. One. 12 (6), e0179036. 10.1371/journal.pone.0179036 28628611 PMC5476241

[B40] Ma R. Wang Y. Huang L. Zhao S. Li L. Yin M. (2021). Effects of Different Salinity on the Transcriptome and Antibiotic Resistance of Two Vibrio Parahaemolyticus Strains Isolated from *Penaeus Vannamei* Cultured in Seawater and Freshwater Ponds. J. Fish. Dis. 44 (12), 2055–2066. 10.1111/jfd.13520 34496040

[B41] Malik A. Kim C.-B. (2021). Role of Transportome in the Gills of Chinese Mitten Crabs in Response to Salinity Change: a Meta-Analysis of RNA-Seq Datasets. Biology 10 (1), 39. 10.3390/biology10010039 33430106 PMC7827906

[B42] Mao H. Huang S. Wang Z. Zhou L. Wang C. (2014). Molecular Cloning and Expression Analysis of Na^+^∼/K^+^∼-ATPase Alpha1 Gene in Chinese Mitten Crab (*Eriocheir Sinensis*). J. Agric. Biol. 22 (3), 343–350. 10.3969/j.issn.1674-7968.2014.03.010

[B43] McNamara J. C. Freire C. A. Torres A. H. Faria S. C. (2015). The Conquest of Fresh Water by the Palaemonid Shrimps: an Evolutionary History Scripted in the Osmoregulatory Epithelia of the Gills and Antennal Glands. Biol. J. Linn. Soc. Lond 114 (3), 673–688. 10.1111/bij.12443

[B44] Mitchell R. T. Henry R. P. (2014). Carbonic Anhydrase Induction in Euryhaline Crustaceans Is Rate-Limited at the Post-transcriptional Level. Comp. Biochem. Physiology Part A Mol. Integr. Physiology 169, 15–23. 10.1016/j.cbpa.2013.12.004 24333600

[B45] Paital B. Chainy G. B. N. (2010). Antioxidant Defenses and Oxidative Stress Parameters in Tissues of Mud Crab (*Scylla serrata*) with Reference to Changing Salinity. Comp. Biochem. Physiology Part C Toxicol. Pharmacol. 151 (1), 142–151. 10.1016/j.cbpc.2009.09.007 19796708

[B46] Pan L. Q. Jiang L. X. Miao J. J. (2005). Effects of Salinity and pH on Immune Parameters of the White Shrimp. Litopenaeus vannameiJ. Shellfish. Res. 24 (4), 1223–1228. 10.2983/0730-8000(2005)24

[B47] Pierrot C. Pequeux A. Thuet P. (1995). Perfusion of Gills Isolated from the Hyper-Hyporegulating Crab Pachygrapsus Marmoratus (Crustacea, Decapoda): Adaptation of a Method. Archives Physiology Biochem. 103 (4), 401–409. 10.3109/13813459509047129 8548473

[B48] Pongsomboon S. Udomlertpreecha S. Amparyup P. Wuthisuthimethavee S. Tassanakajon A. (2009). Gene Expression and Activity of Carbonic Anhydrase in Salinity Stressed Penaeus monodon. Comp. Biochem. Physiol. A Mol. Integr. Physiol. 152 (2), 225–233. 10.1016/j.cbpa.2008.10.001 18950726

[B49] Réalis-Doyelle E. Schwartz J. Dubos M. P. Favrel P. (2021). Molecular and Physiological Characterization of a Crustacean Cardioactive Signaling System in a Lophotrochozoan - the Pacific Oyster (Crassostrea gigas): a Role in Reproduction and Salinity Acclimation. J. Exp. Biol. 224 (10), jeb241588. 10.1242/jeb.241588 34028518

[B50] Riesgo A. Andrade S. C. S. Sharma P. P. Novo M. Pérez-Porro A. R. Vahtera V. (2012). Comparative Description of Ten Transcriptomes of Newly Sequenced Invertebrates and Efficiency Estimation of Genomic Sampling in Non-model Taxa. Front. Zool. 9 (1), 33. 10.1186/1742-9994-9-33 23190771 PMC3538665

[B51] Rivera-Ingraham G. A. Barri K. Boël M. Farcy E. Charles A.-L. Geny B. (2015). Osmoregulation and Salinity-Induced Oxidative Stress: Is Oxidative Adaptation Determined by Gill Function? J. Exp. Biol. 219 (1), 80–89. 10.1242/jeb.128595 26567341

[B52] Shi J. Fu M. Zhao C. Zhou F. Yang Q. Qiu L. (2016). Characterization and Function Analysis of Hsp60 and Hsp10 under Different Acute Stresses in Black Tiger Shrimp, *Penaeus monodon* . Cell Stress Chaperones 21, 295–312. 10.1007/s12192-015-0660-6 26637414 PMC4786529

[B53] Simão F. A. Waterhouse R. M. Ioannidis P. Kriventseva E. V. Zdobnov E. M. (2015). BUSCO: Assessing Genome Assembly and Annotation Completeness with Single-Copy Orthologs. Bioinformatics 31 (19), 3210–3212. 10.1093/bioinformatics/btv351 26059717

[B54] Sun S. M. Guo Z. B. Fu H. T. Ge X. P. Zhu J. Gu Z. M. (2018). Based on the Metabolomic Approach the Energy Metabolism Responses of Oriental River Prawn *Macrobrachium Nipponense* Hepatopancreas to Acute Hypoxia and Reoxygenation. Front. Physiol. 9, 11. 10.3389/fphys.2018.00076 29686619 PMC5900017

[B55] Sun S. Xuan F. Fu H. Ge X. Zhu J. Qiao H. (2016). Molecular Characterization and mRNA Expression of Hypoxia Inducible Factor-1 and Cognate Inhibiting Factor in *Macrobrachium Nipponense* in Response to Hypoxia. Comp. Biochem. Physiology Part B Biochem. Mol. Biol. 196-197, 48–56. 10.1016/j.cbpb.2016.02.002 26883381

[B56] Sun S. Xuan F. Fu H. Zhu J. Ge X. Gu Z. (2015). Transciptomic and Histological Analysis of Hepatopancreas, Muscle and Gill Tissues of Oriental River Prawn (*Macrobrachium Nipponense*) in Response to Chronic Hypoxia. BMC Genomics 16 (1), 491. 10.1186/s12864-015-1701-3 26138936 PMC4490754

[B57] Sun S. Zhu M. Pan F. Feng J. Li J. (2020). Identifying Neuropeptide and G Protein-Coupled Receptors of Juvenile Oriental River Prawn (*Macrobrachium Nipponense*) in Response to Salinity Acclimation. Front. Endocrinol. 11, 623. 10.3389/fendo.2020.00623 PMC7506046 33013701

[B58] Tong R. Pan L. Zhang X. Li Y. (2022). Neuroendocrine‐immune Regulation Mechanism in Crustaceans: A Review. Rev. Aquacult. 14, 378–398. 10.1111/raq.12603

[B59] Torres G. Giménez L. Anger K. (2011). Growth, Tolerance to Low-Salinity, and Osmoregulation in Decapod Crustacean Larvae. Aquat. Biol. 12, 249–260. 10.3354/ab00341

[B60] Tseng Y.-C. Hwang P.-P. (2008). Some Insights into Energy Metabolism for Osmoregulation in Fish. Comp. Biochem. Physiology Part C Toxicol. Pharmacol. 148 (4), 419–429. 10.1016/j.cbpc.2008.04.009 18539088

[B61] Wang H. Tang L. Wei H. Lu J. Mu C. Wang C. (2018). Transcriptomic Analysis of Adaptive Mechanisms in Response to Sudden Salinity Drop in the Mud Crab, *Scylla Paramamosain* . Bmc. Genomics. 19, 421. 10.1186/s12864-018-4803-x 29855258 PMC5984308

[B62] Wang T. Shan H.-W. Geng Z.-X. Yu P. Ma S. (2020). Dietary Supplementation with Freeze-Dried Ampithoe Sp. Enhances the Ammonia-N Tolerance of *Litopenaeus Vannamei* by Reducing Oxidative Stress and Endoplasmic Reticulum Stress and Regulating Lipid Metabolism. Aquac. Rep. 16, 100264. 10.1016/j.aqrep.2019.100264

[B63] Wang W.-N. Zhou J. Wang P. Tian T.-T. Zheng Y. Liu Y. (2009). Oxidative Stress, DNA Damage and Antioxidant Enzyme Gene Expression in the Pacific White Shrimp, *Litopenaeus Vannamei* when Exposed to Acute pH Stress. Comp. Biochem. Physiology Part C Toxicol. Pharmacol. 150, 428–435. 10.1016/j.cbpc.2009.06.010 19573624

[B64] Wei J. Tian L. Wang Y. Yu L. Zhu X. (2021). Effects of Salinity, Photoperiod, and Light Spectrum on Larval Survival, Growth, and Related Enzyme Activities in the Giant Freshwater Prawn, Macrobrachium Rosenbergii. Aquaculture 530, 735794–738486. 10.1016/j.aquaculture.2020.735794

[B65] Williams W. D. (2001). Anthropogenic Salinisation of Inland Waters. Hydrobiologia 466, 329–337. 10.1023/A:1014598509028

[B66] Yang Z. Zhou J. Wei B. Cheng Y. Zhang L. Zhen X. (2019). Comparative Transcriptome Analysis Reveals Osmotic-Regulated Genes in the Gill of Chinese Mitten Crab (*Eriocheir Sinensis*). PLOS. one 14 (1), e0210469. 10.1371/journal.pone.0210469 30629688 PMC6328174

[B67] Ye L. Jiang S. Zhu X. Yang Q. Wen W. Wu K. (2009). Effects of Salinity on Growth and Energy Budget of Juvenile Penaeus monodon. Penaeus monodonAquaculture 290 (1), 140–144. 10.1016/j.aquaculture.2009.01.028

[B68] Ye L. Jiang S. Zhu X. Yang Q. Wen W. Wu K. (2009). Effects of Salinity on Growth and Energy Budget of Juvenile *Penaeus monodon* . Aquaculture 290, 140–144. 10.1016/j.aquaculture.2009.01.028

[B69] Zhang D. Wang F. Dong S. Lu Y. (2015). De Novo assembly and Transcriptome Analysis of Osmoregulation in *Litopenaeus Vannamei* under Three Cultivated Conditions with Different Salinities. Gene 578 (2), 185–193. 10.1016/j.gene.2015.12.026 26691500

[B70] Zhang K. F. Zhang Z. P. Chen Y. (2007). Antioxidant Defense System in Animals. J. Zool. 42 (2), 153–116. 10.13859/j.cjz.2007.02.035

[B71] Zhao C. Fu H. Sun S. Qiao H. Zhang W. Jin S. (2018). A Transcriptome Study on *Macrobrachium Nipponense* Hepatopancreas Experimentally Challenged with White Spot Syndrome Virus (WSSV). PLoS. One. 13 (7), e0200222. 10.1371/journal.pone.0200222 29979781 PMC6034857

