# Supplementary material for: Transcriptome Analysis to Study the Molecular Response in the Gill and Hepatopancreas Tissues of Macrobrachium nipponense to Salinity Acclimation
Source: Front Physiol. 2022 May 25;13:926885. doi: 10.3389/fphys.2022.926885 (PMC9176394; doi:10.3389/fphys.2022.926885)
Supplement: Supplementary file 1 [file DataSheet1.docx]

**Transcriptome analysis to study the molecular response in the gill and hepatopancreas tissues of *Macrobrachium nipponense* to salinity acclimation**

Cheng Xue^a,b^, Kang Xu^a,b^, Yiting Jin^a,b^, Chao Bian^c^, Shengming Sun ^a,b*^

^a^ Key Laboratory of Exploration and Utilization of Aquatic Genetic Resources, Shanghai Ocean University, Ministry of Education, Shanghai 201306, PR China

^b^ International Research Center for Marine Biosciences at Shanghai Ocean University, Ministry of Science and Technology, Shanghai 201306, PR China

^c^ Shenzhen Key Lab of Marine Genomics, Guangdong Provincial Key Lab of Molecular Breeding in Marine Economic Animals, BGI Academy of Marine Sciences, BGI Marine, BGI, Shenzhen 518083, PR China

*Corresponding author: Shengming Sun

Key Laboratory of Exploration and Utilization of Aquatic Genetic Resources, Shanghai Ocean University, Ministry of Education, Shanghai 201306, PR China E-mail: sunshengming621416@163.com.

**Table S1. Statistics of de novo transcriptome assembly and annotation**

| Total unigenes number | 162,250 |
| --- | --- |
| Total unigenes length | 187,598,373 |
| Total isoform number | 248,326 |
| Total isoform length | 368,955,322 |
| Average Unigene length: | 1156.23 |
| Largest unigene: | 19,804 |
| N50: | 1456 |

N50: Transcripts sorted by length from long to short, for which the sum of the length of transcripts was not less than 50% of the total length of mosaic transcripts.

**Table S2. Length distribution of the unigenes from the de novo assembly**

| Unigene length | Unigene number | Percent(%) |
| --- | --- | --- |
| 1-400 | 12,280 | 7.56 |
| 401-600 | 39,874 | 24.58 |
| 601-800 | 28,012 | 17.26 |
| 801-1000 | 19,867 | 12.24 |
| 1001-1200 | 14,022 | 8.64 |
| 1201-1400 | 10,358 | 6.39 |
| 1401-1600 | 7689 | 4.78 |
| 1601-1800 | 5688 | 3.52 |
| 1801-2000 | 4525 | 2.63 |
| 2001-2400 | 6246 | 3.85 |
| 2401-2800 | 3932 | 2.46 |
| 2801-3200 | 2773 | 1.74 |
| 3201-3600 | 1850 | 1.18 |
| 3601-4000 | 1222 | 0.76 |
| 4001-5000 | 1923 | 1.19 |
| 5001-6000 | 972 | 0.59 |
| 6001-7000 | 486 | 0.29 |
| 7001-8000 | 261 | 0.18 |
| 8001-9000 | 132 | 0.08 |
| 9001-10000 | 47 | 0.03 |
| 10001-20000 | 91 | 0.05 |
| ALL | 162,250 | 100 |

**Table** **S3. Statistics of the annotation results for *Macrobrachium* *nipponense***

| Sample | All | NCBI nr | Swiss-port | GO | KEGG | COG |
| --- | --- | --- | --- | --- | --- | --- |
| No. of unigenes | 162,250 | 51,172 | 34,276 | 10,002 | 27,554 | 16,078 |
| % of unigenes | 100 | 31.54 | 21.13 | 6.16 | 16.98 | 9.89 |

**Table S4. GO enrichment analyses of DEGs (p value ≤ 0.05)**

| Sample | GO enrich num | BP | CC | MF |
| --- | --- | --- | --- | --- |
| LG *vs.* FG | 578 | 346 | 94 | 138 |
| HG *vs.* FG | 286 | 139 | 38 | 109 |
| LH *vs.* FH | 176 | 77 | 48 | 51 |
| HH *vs.* FH | 221 | 52 | 73 | 96 |
| Sum | 1261 | 614 | 253 | 394 |

**Table S5. SSR analysis of transcriptome data of *Macrobrachium* *nipponense***

| Searching item | Numbers |
| --- | --- |
| Total number of sequences examined | 194,085 |
| Total size of examined sequences (bp) | 175,702,315 |
| Total number of identified SSRs: | 73,804 |
| Number of SSR containing sequences | 53,006 |
| Number of SSR containing sequences | 14,923 |
| Number of SSRs present in compound formation | 6,033 |

**Table S6. DEGs potentially associated with salinity accumulation of** ***Macrobrachium* *nipponense***

| Feature ID | Title | Gene function | Log_2_(FC)  (HG *vs.* FG) | Log_2_(FC)  (LG *vsF*G) | log_2_(FC)  (HH *vs.* FH) | log_2_(FC)  (LH *vs.* FH) |
| --- | --- | --- | --- | --- | --- | --- |
| DN101611_c0_g1 | ATP synthase (ATPase) | Ion transport | 11.67 | 11.67 | 10.12 | 4.28 |
| DN182443_c0_g1 | V-type proton ATPase subunit E-likeC(VHA E) | Ion transport | 11.12 | 4.920 | 8.93 | 2.52 |
| DN104533_c0_g1 | Sodium potassium-transporting ATPase (NKA) | Ion transport | 12.33 | 0.45 | 4.86 | 1.35 |
| DN105331_c0_g1 | Sarco/endoplasmic reticulum Calcium-ATPase isoform 1 (SERCA1) | Ion transport | 19.72 | 16.75 | 12.70 | 7.88 |
| DN209426_c0_g4 | carbonic anhydrase (CA) | Acid-base balance | 9.88 | 11.50 | 9.12 | 8.18 |
| DN127644_c0_g1 | glucose transporter 2(GLUT2) | [Glucose](javascript:;) [metabolism](javascript:;) | 15.28 | 11.39 | 11.72 | 7.12 |
| DN203590_c1_g2 | glucose transporter 1(GLUT1) | [Glucose](javascript:;) [metabolism](javascript:;) | 11.66 | 10.22 | 9.49 | 12.92 |
| DN43844_c0_g1 | mitochondrial manganese superoxide dismutase (mMnSOD) | Antioxidation | -9.75 | -2.02 | -15.31 | -2.45 |
| DN63332_c0_g1 | copper/zinc superoxide dismutase (CuZnSOD) | Antioxidation | -14.00 | -1.93 | -4.64 | -2.80 |
| DN203024_c0_g1 | Catalase (CAT) | Immune Response | 15.53 | 11.24 | 16.51 | 8.18 |
| DN4387_c0_g1 | glutathione peroxidase 3 (GPx3) | Immune Response | 18.44 | 8.77 | 19.98 | 8.16 |
| DN137974_c0_g1 | heat shock protein 70 (HSP70) | Immune Response | 11.87 | 7.63 | 16.58 | 6.28 |
| DN200338_c2_g1 | heat shock protein 90 (HSP90) | Immune Response | 7.70 | 3.88 | 7.11 | 3.20 |
| DN95010_c0_g1 | L-lactate dehydrogenase (LDH) | Glycolysis | 9.87 | 1.65 | 7.25 | 1.84 |

Note: Log_2_(FC) means fold change of expression level of the comparative group.

**Figure S1**


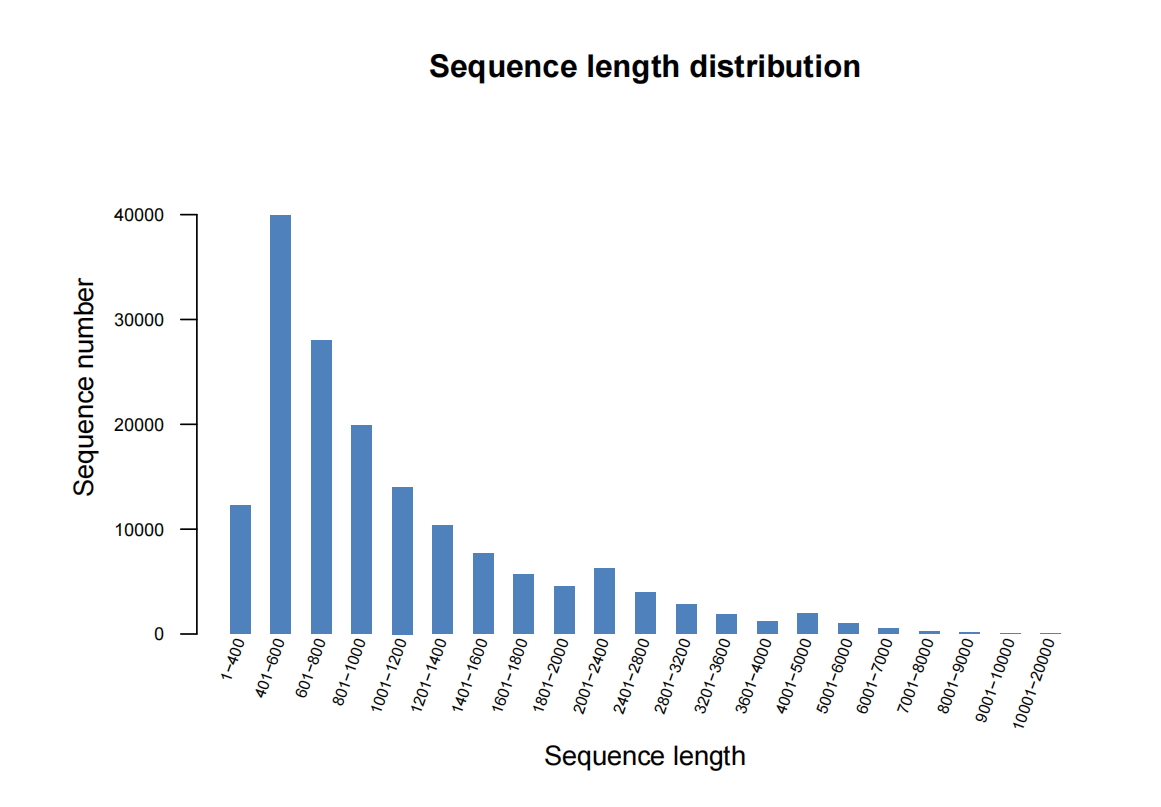


**Figure S1 Assembly sequence length distribution**

**Figure S2**

**A:DH vs FH**   **B：HH vs FH**


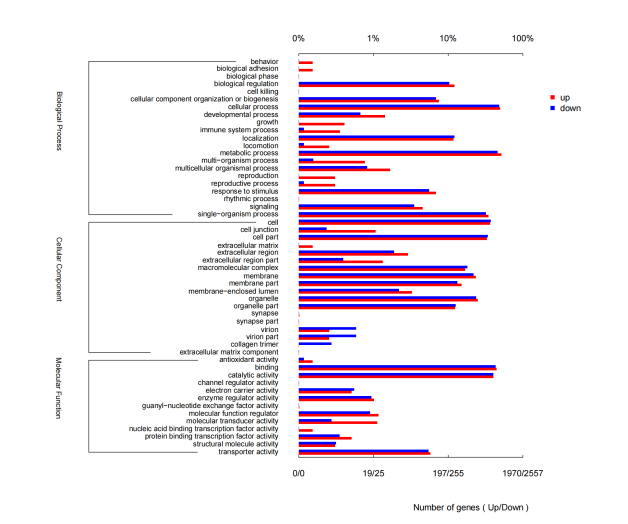

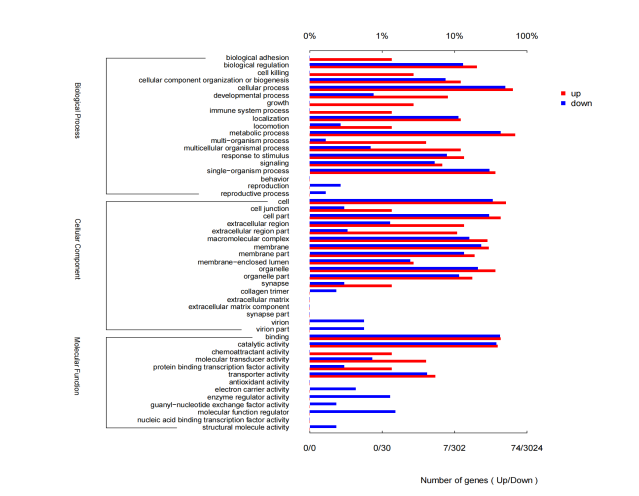


**C:LG vs FG**   **D:HG vs FG**


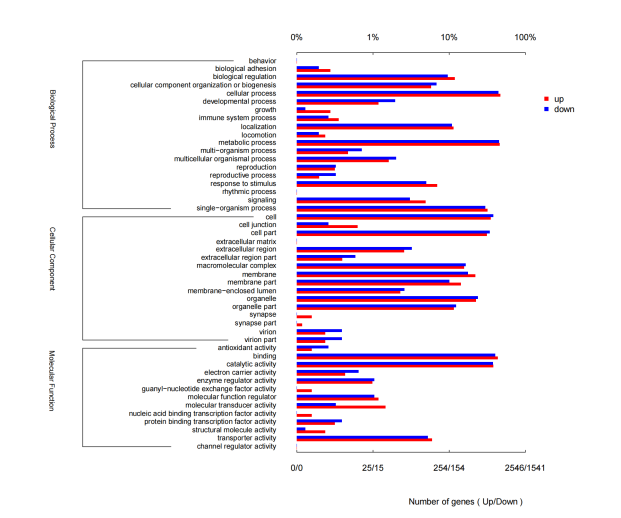

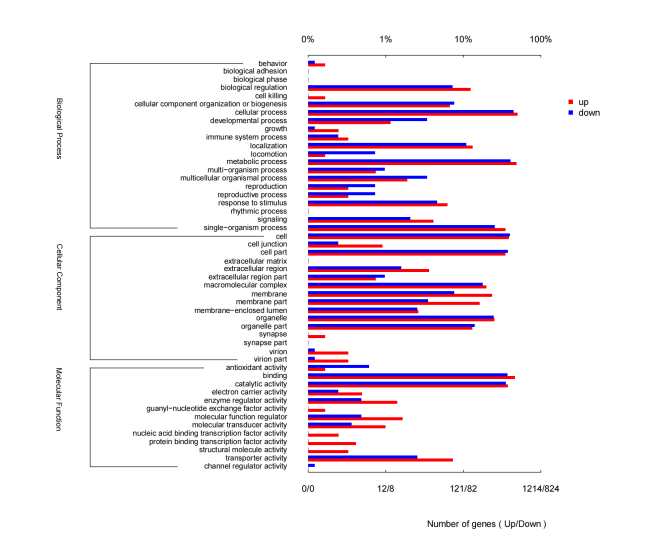


**Figure S2 The GO classification analyses of DEGs.**

**Figure S3**


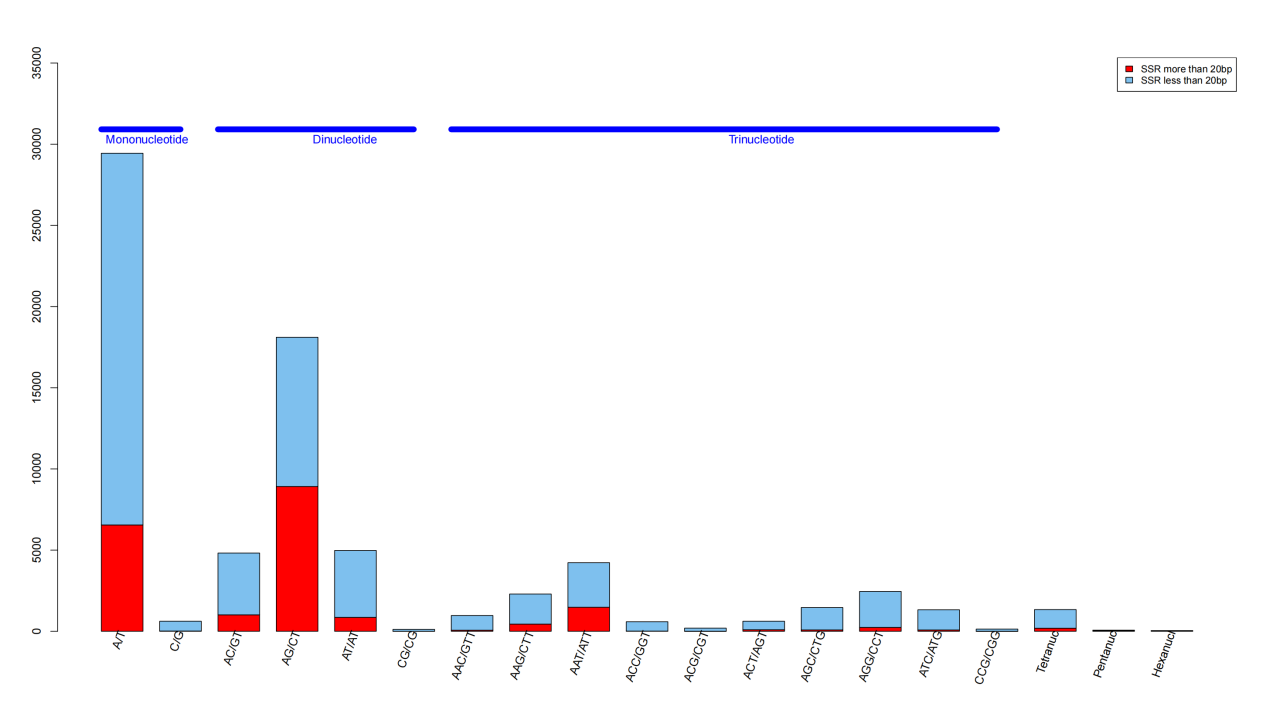


**Figure S3 Statistics of SSR distribution**
